# An epidemiological survey on low birth weight infants in China and analysis of outcomes of full-term low birth weight infants

**DOI:** 10.1186/1471-2393-13-242

**Published:** 2013-12-26

**Authors:** Yi Chen, Guanghui Li, Yan Ruan, Liying Zou, Xin Wang, Weiyuan Zhang

**Affiliations:** 1 Department of Obstetrics, Beijing Obstetrics and Gynecology Hospital, Capital Medical University, Beijing 100026, China

**Keywords:** Low birth weight (LBW), Incidence, High-risk pregnancy, Perinatal outcomes

## Abstract

**Background:**

Low birth weight (LBW) is one of the leading causes of adverse perinatal outcomes and is closely related to neonatal disease and death. The incidence of LBW has been increasing. The aim of this study was to investigate the current incidence rate and factors affecting low birth weight infants and perinatal outcomes of full-term low birth weight infants in mainland China.

**Methods:**

This paper describes a retrospective analysis of children born in 39 hospitals of different types in 14 different provinces, municipalities, and autonomous regions in seven districts within China throughout 2011. The data were first collected in hardcopy format and then entered into computer network databases. Data covering a total of 112,441 cases were collected. Cases were excluded if data were incomplete and in the case of miscarriage before 24 weeks of gestation, multiple pregnancies, or induction of labor due to fetal malformation, intrauterine death, and other reasons, leaving a total of 101,163 cases. SPSS 18.0 and SAS 9.2 statistical packages were used to analyze the collected data.

**Results:**

According to this research, the incidence of LBW in mainland China was 6.1%, which is higher than the 5.87% reported in 2000, and it varied across different areas. The incidence of LBW was significantly higher in tertiary care hospitals than in secondary care hospitals. LBW was found to be associated with maternal age of less than 20 years, low level of maternal education, previous histories of adverse pregnancies, and with pregnancy comorbidities and complications, such as hypertensive disorders during pregnancy, anemia, oligohydramnios, premature rupture of membranes, and gestational diabetes. The rates of stillbirths, severe neonatal asphyxia, and deaths among full-term LBW infants were 2.42%, 0.83%, and 3.49%, respectively. The rates of stillbirths and neonatal deaths among full-term LBW infants born by caesarean section were 0.5% and 1.0%, respectively, which was lower than vaginal delivery.

**Conclusions:**

The incidence of LBW has increased in China. LBW is a leading cause of adverse pregnancy outcomes. Health care during pregnancy and management of high-risk factors for LBW may reduce the incidence of LWB and the death rate of LBW infants.

## Background

LBW refers to birth weights below 2500 grams. It is one of the major causes of adverse perinatal outcomes and death. A study has shown that neonatal death among infants weighing 1500–2500 grams is 20 times higher than among infants of normal weight [1]. LBW is also a leading cause of childhood diseases and deaths and is closely related to future hypertension, diabetes, and other metabolic diseases [2]. With socio-economic development, progress in modern medicine, and improvements in the treatment of high-risk infants, the incidence of premature and LBW infants has increased. A study carried out in the U.S. showed that the incidence of LBW in the New York area had increased from 7.7% in 1996 to 8.2% in 2009 [3]. The incidence of LBW is even higher in developing countries. For example, it was reported to be 17.1% in Ethiopia and as high as 19.3% in India [4,5]. Therefore, LBW has drawn attention as a public health issue, especially in developing countries such as China. In 2000, the average incidence of LBW across 11 provinces was reported to be 5.87% [6]. However, no similar surveys have been conducted in China during the past 10 years. The purpose of this study was to assess the current epidemiological status of LBW in mainland China and the relevance of specific factors such as maternal medical history, pregnancy comorbidities and complications, and the method of delivery to the final outcome.

## Methods

The current report was performed in mainland China. It is a multi-centered, large sample cross-sectional study. The present paper discribes a retrospective analysis that was performed on 112,441 babies delivered in 2011 and their mothers. Clinical data were obtained from 14 provinces, municipalities and autonomous regions within China (Beijing, Shanghai, Jilin, Liaoning, Jiangsu, Sichuan, Shanxi, Hubei, Guangdong, Hebei, Inner Mongolia, Shandong, Shanxi, and Xinjiang), covering 39 hospitals of different levels. The hospitals included 12 tertiary care general hospitals, 7 tertiary care specialty hospitals, 8 secondary care general hospitals, and 12 secondary care specialty hospitals (in Chinese hospital classification system, tertiary care is the most speciallized, and primary care is the least specialized [7]), covering mainland China but not Hong Kong, Taiwan, or Macao. Clinical data included the maternal medical history, pregnancy comorbidities and complications, the results of examinations conducted during pregnancy, the method of delivery, the infant’s APGAR scores, and maternal and neonatal outcomes.

Based on geographical distribution, functions, and care services, Chinese hospitals are classified as primary, secondary, and tertiary care hospitals. The primary care facilities include hospitals and community-based health care facilities that provide disease prevention, health care, rehabilitation, and other basic services to communities. The majority of primary care hospitals only provide low-level care and do not have an obstetric department. They are not equipped to perform cesarean sections, and there is no emergency obstetric or neonatal care service. Medical data collected at these hospitals are often incomplete. Therefore, primary care hospitals were not included in the present study. All delivery data were collected from tertiary care general hospitals, tertiary care specialty hospitals, secondary care general hospitals, and secondary care specialty hospitals. These data were considered representative of the overall distribution of LBW and delivery methods in China.

**Figure 1:**
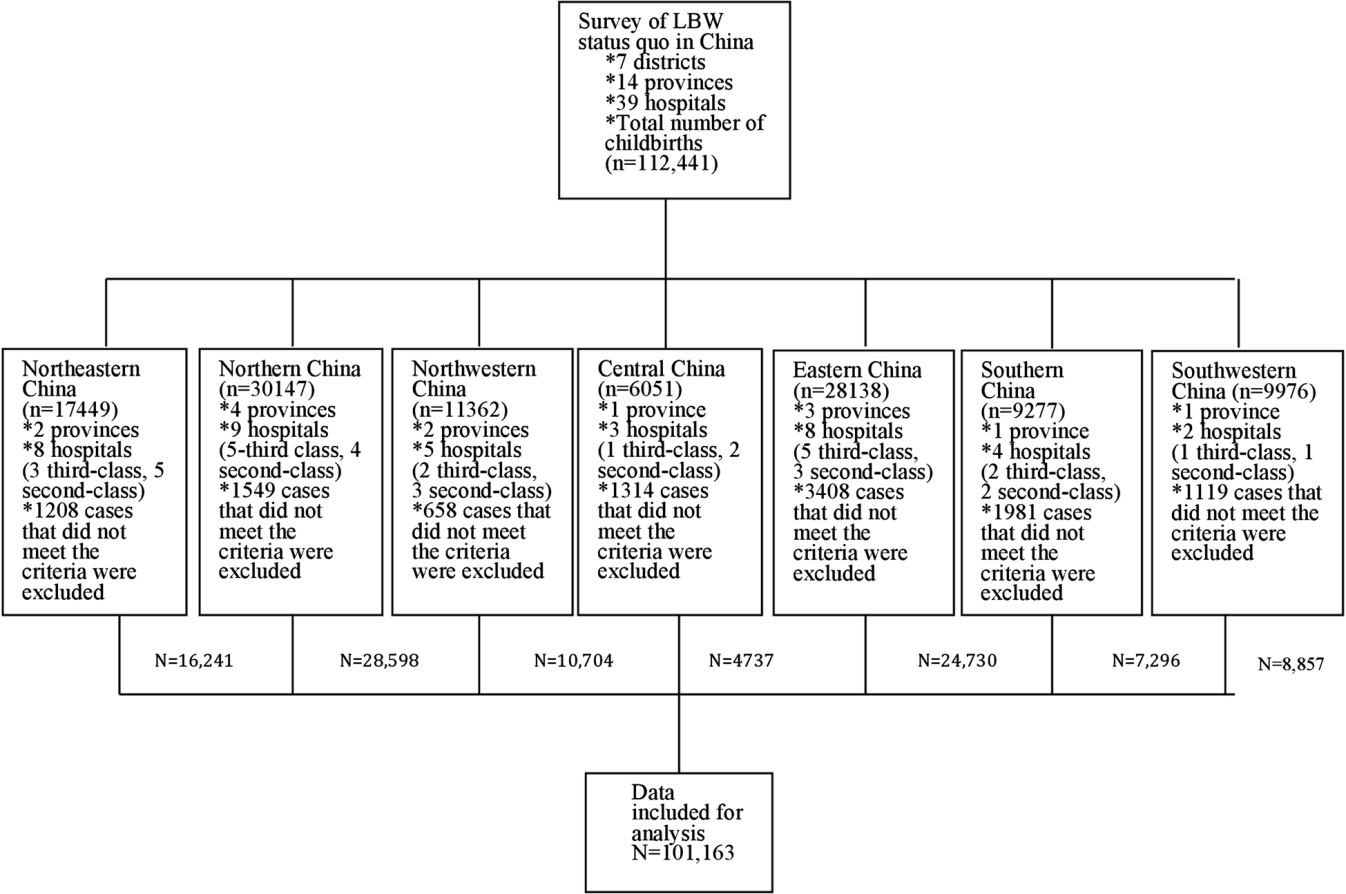
A retrospective analysis that was performed on 112,441 babies, covering 39 hospitals of diferent levels in mailand China.

**Figure 2:**
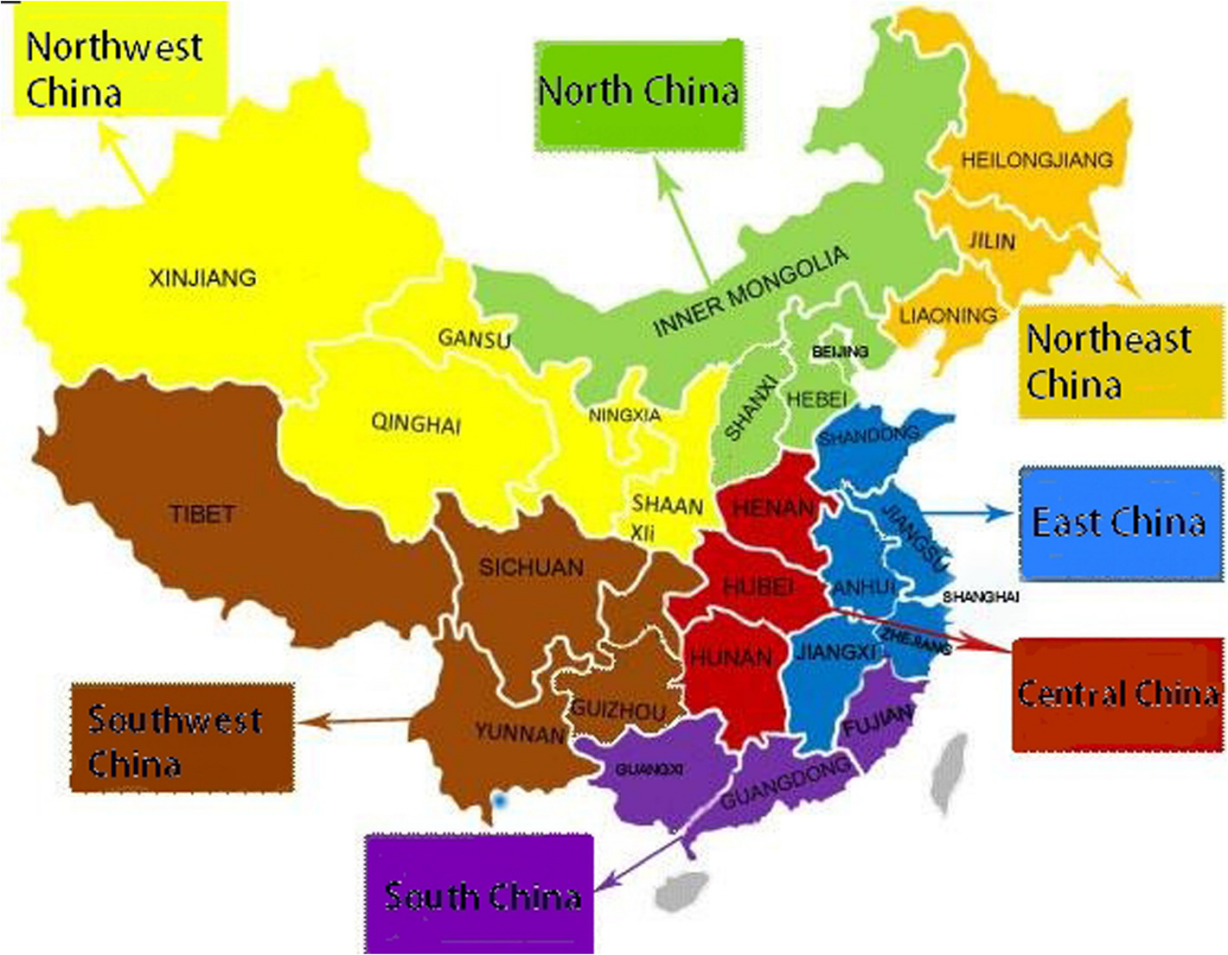
**The map of Chinese administrative division.** Data were obtained from 14 provinces, municipalities and autonomous regions.

To ensure patient privacy, data did not include the mother’s name, phone number, home address, or other personal information. In total, 939 miscarriages before 24 weeks of gestation or of induction of labor due to fetal malformations, intrauterine death, or other reasons and 10,339 cases of multiple pregnancies were excluded. Distributions of data included for analysis are shown in Figures 1 and 2.

Questionnaire: The questionnaire included the mother’s age, ethnic group, level of education, marital status, employment status, medical history, parity, history of pregnancy, pregnancy comorbidity and complications, method of delivery, and maternal and neonatal outcomes, and so on. The questionnaire was designed by obstetric and statistical experts and finalized after many discussions regarding its feasibility (Additional file 1).

Training of investigators: The head of each sub-center in each province, municipality, or autonomous region accompanied 2–3 investigators to attend face-to-face training on questionnaire entry and completion. Instructions for completing the questionnaire were also sent out to the investigators.

Data entry: Investigators from each province, municipality, and autonomous region were responsible for training personnel for data entry. Data were first entered in hardcopy format and then entered into computer network databases.

Quality control: In each sub-center 1–2 specialized personnel were trained in data quality control, and were responsible for their entire region. After the data were sent to the survey headquarters, specialized personnel at the headquarters were responsible for the second round of quality control assessment.

Data collection: Data were collected and entered into to a computer network database. Case collection and hardcopy data entry were carried out from January-April 2012. Then, data were entered into network database from May-June 2012, and data quality control was carried out during the same period. Each participating hospital was responsible for its own case collection and data entry, and all personnel that participated in data entry received training beforehand. Data included birth outcomes of each hospital throughout 2011.

### Ethical approval

The study was approved by the Ethics Committee of Beijing Obstetrics and Gyenacology Hospital in accordance with the Helsinki Declaration and a written informed consent form was obtained from each adult participant.

### Main concepts and abbreviations

Low birth weight (LBW): Neonates with birth weights lower than 2500 grams [1].

Preterm birth (PTB): Delivery after at least 28 weeks (≥196 days) gestation but no more than 37 weeks (≤258 days) gestation [8]. The last menstrual period (LMP) method was used to calculate the gestational age of each participating neonate. If LMP was unknown or if the mother’s menstrual period was irregular, gestational age was determined by fetal size as indicated by B-ultrasound early during pregnancy or fetal size and growth rate as indicated by ultrasound during mid-trimester.

Gestational diabetes mellitus (GDM): GDM refers to various degrees of abnormal glucose tolerance occurring or found for the first time during pregnancy. Blood glucose threshold values for fasting OGTT and at 1 h, 2 h, were 5.1, 10.0, 8.5 mmol/L (92, 180, 153 mg/dl), respectively. If any measurement reached or exceeded these threshold values, the patient was diagnosed with GDM [9].

### Statistical methods

Data were analyzed using SPSS 18.0 and SAS 9.2 statistical analysis software packages. For qualitative data, the chi-square test was performed; for quantitative data ANOVA was performed; for correlation analysis, multivariate unconditional logistics regression analysis was performed. P < 0.05 was considered statistically significant.

## Results

### Incidence of LBW

In total, 101,163 deliveries ≥24 weeks of gestation were included in the present study. Of these, there were 6135 cases of LBW, resulting in an incidence rate of 6.1%. Out of all cases of LBW, there were 1863 births ≥37 weeks, and the incidence of full-term LBW was 2.0% (1863/93874). Based on gestational age, PTB LBW accounted for 69.6% of all LBW cases, and full-term LBW accounted for 30.4%.

The incidence of LBW was highest in Southwestern China (9.4%) and lowest in central China (2.5%). The LBW incidences in all other regions were 5.1-9.0%. There was a significant difference in the incidence of LBW in different regions (P < 0.001). The incidences of LBW in Southwestern China and northeastern China were significantly higher than in other regions. The incidence rates in Beijing and Shanghai, which are large, thriving cities, were 4.9% and 4.2%, respectively. The incidence of LBW in Xinjiang, which is a remote and highly rural region, was 12.9%.

Among the 101,163 pregnant women, the incidence of LBW in infants delivered in teriary care hospitals was 7.1% (5523/77338), and the incidence of LBW in infants delivered in secondary care hospitals was 2.6% (612/23825). The incidence of LBW in teriary care hospitals was significantly higher than in secondary care hospitals (P < 0.001).

### Risk factors for LBW

Unconditional logistics regression analysis was performed on possible factors related to LBW occurrence, including maternal age, maternal height, pre-pregnancy weight, body mass index (BMI), pregnancy weight gain, parity, previous history of miscarriage, history of fetal death and stillbirth, drinking, smoking, education level, gestational hypertensive disorders, gestational diabetes, premature rupture of membranes, abnormal amniotic fluid volume, anemia, pregnancy with uterine leiomyomas, heart disease during pregnancy, and other factors. Prescreening using univariate logistics regression analysis followed by unconditional binary logistic regression analysis showed that maternal age ≤20 years, low educational level, history of one or more spontaneous abortions, history of one or more stillbirths, pregnancy-induced hypertension, anemia, premature rupture of membranes, oligohydramnios, preterm birth, and BMI <18.5 were risk factors. In cases of GDM, weight gain was a protective factor (Table 1).

**Table 1:** Analysis of factors influencing LBW

**Variables**		**Normal**		**LBW**		**P**	**OR**	**95% C.I. of OR**	
		**N**	**%**	**N**	**%**			**Lower limit**	**Upper limit**
Maternal age	21-34 years old	83301	93.5	5414	6.5	0.020	1		
	≤20 years old	1767	90.6	166	9.4	0.097	1.332	1.046	1.695
	≥35 years old	9960	94.4	555	5.6	0.000	0.097	0.789	1.020
Maternal educational level	Illiterate	1715	90.8	157	9.2		1		
	Tertiary or higher Technical	46970	94.3	2691	5.7	<0.001	0.259	0.121	.551
	Secondary school, high school, or higher	30079	93.7	1906	6.3	0.004	0.332	0.156	.707
	Middle school or below	16264	91.5	1381	8.5	0.001	0.293	0.137	.624
Parity	0	9940	93.0	599	6.0		1		
	1	68263	93.9	4155	6.1	0.055	1.561	0.991	2.459
	2 or more	16825	91.8	1381	8.2	0.785	0.927	0.537	1.599
Spontaneous miscarriages	0	92267	93.6	5898	6.4		1		
	1 or more	2761	91.4	237	8.6	0.023	1.362	1.043	1.777
Stillbirths	0	94650	93.6	6073	6.4		1		
	1 or more	378	83.6	62	16.4	<0.001	2.446	1.479	4.044
GDM	No	90678	93.5	5916	6.5				
	GDM	4218	94.4	239	5.6	0.935	1.010	.797	1.280
	DM complicated with pregnancy	132	81.1	25	18.9	0.011	0.263	.094	.733
Hypertensive disorders in pregnancy	No	90085	94.8	4712	5.2		1		
	Yes	4943	71.2	1423	28.8	<0.001	5.031	4.325	5.853
Anemia	No	89039	93.7	5618	6.3		1		
	Yes	5989	91.4	517	8.6	0.032	1.230	1.017	1.488
Premature rupture of membranes	No	80983	94.5	4479	5.5				
	Yes	14045	88.3	1656	11.7	0.027	1.154	1.016	1.311
Abnormal amniotic fluid volume	None	90290	92.5	5666	6.3		1		
	Too much amniotic fluid	1162	91.2	102	8.8	0.370	0.809	.509	1.286
	Too little amniotic fluid	3576	89.7	367	10.3	<0.001	2.068	1.659	2.578
PTB	No	93874	97.5	1863	2.5				
	Yes	1154	38.4	4272	61.6	<0.001	46.246	41.484	51.555
BMI	18.5 ~ 24	39929	94.5	2312	5.5		1		
	<18.5	6872	92.7	538	7.3	<0.001	1.637	1.415	1.893
	24 ~ 27.9	8263	93.7	554	6.3	0.026	0.840	.721	.980
	≥28	2110	93.3	151	6.7	0.001	0.620	.466	.826
Weight gain	<11	13382	92.1	1146	7.9		1		
	11-13.9	11242	94.0	716	6.0	<0.001	0.764	.660	.884
	14-16.9	13318	94.3	798	5.7	<0.001	0.721	.624	.831
	≥17	15637	94.2	963	5.8	<0.001	0.628	.545	.723
Height						<0.001	0.951	.941	.961
Constant						<0.001	1123.42		

### Method of delivery of LBW infants and prognosis

#### Comparison of delivery methods

In the present study, LBW infants were divided into two groups, full-term (≥37 weeks) and preterm (<37 weeks), to control for the effects of PTB on pregnancy outcome. Delivery methods used in full-term LBW infants and in full-term infants with normal birth weight were compared. Full-term LBW infants were more likely to have been delivered by caesarian section (61.1%) than full-term infants with normal birth weight (52.9%). The difference was statistically significant (P < 0.001). Of all full-term LBW infants, 41.1% were delivered by emergency cesarean sections. In contrast, only 31.1% of full-term infants with normal birth weight required emergency cesarean sections (P < 0.001).

We compared pregnancy outcomes, including complications during childbirth and outcomes of neonatal infants, and full-term LBW infants delivered by different methods. Delivery methods included cesarean section, vaginal birth, assisted vaginal delivery, and assisted breech birth. Because of the small number of cases of assisted vaginal delivery and breech birth, comparisons were only made between cesarean section and normal vaginal delivery. There were significantly fewer stillbirths and neonatal deaths among those who underwent a cesarean section than those who underwent vaginal birth (Tables 2 and 3).

**Table 2:** Intrapartum complications among full-term LBW neonates according to mode of delivery

**Mode of delivery**	**Cesarean section**		**Normal vaginal delivery**		***P***
	**n**	**%**	**n**	**%**	
Normal	1028	92.0	619	90.0	
Mild asphyxia	72	6.4	27	3.9	0.039
Severe asphyxia	11	1.0	4	0.6	0.385
Stillbirth	6	0.5	38	5.5	<0.001

**Table 3:** Outcomes of full-term LBW neonates according to mode of delivery

**Mode of delivery**	**Cesarean section**		**Normal vaginal delivery**		***P***
	**n**	**%**	**n**	**%**	
Healthy	1105	97.2	648	91.5	
Complications	16	1.4	6	0.8	0.349
Deformities	5	0.4	6	0.8	0.229
Deaths	11	1.0	48	6.8	<0.001

Analysis of prognosis of full-term LBW infants and infants with normal birth weight showed the occurrence of stillbirth, severe neonatal asphyxia, and neonatal death to be 2.42%, 0.83%, and 3.49%, respectively, among full-term LBW infants and 0.14%, 0.11%, and 0.14%, respectively, among full-term infants with normal birth weight. Differences between the two groups were found to be statistically significant (P < 0.001). Full-term LBW and adverse outcome of perinatal infants were significantly correlated.

## Discussion

According to guidelines published by the World Health Organization (WHO), any neonate with a birth weight below 2500 grams is considered to have LBW. LBW and PTM are leading causes of adverse perinatal outcomes and are closely related to neonatal diseases and deaths. In addition, when LBW infants enter adulthood, they are substantially more likely to suffer from hypertension, diabetes, and other metabolic diseases than individuals of normal birth weight; therefore, increasing the burden on their families and the community [10,11]. Because birth weight can be predictive of the child’s health throughout life, it merits special attention. Throughout the world, there are more than 20 million LBW infants born each year, and 95.6% are born in developing countries. In 2000, the average incidence of LBW in developing countries was 16.5%, which is twice as high as in developed countries [12]. Data collected during the past decade have shown that nearly half of all LBW births throughout the world occur in developing countries in Southern Asia. The incidence of LBW in India is as high as 19.3% [4]. Because the incidence of LBW had been increasing, in 2012, the WHO proposed the goal of achieving a 30% reduction of the number of LBW infants by 2025 [13]. Similarly, the U.S. Department of Health and Human Services has made a goal of reducing the incidence of LBW to 5% [14].

The total incidence of LBW in the present study increased slightly from 5.87% in 2000, but was substantially lower than that reported in India and Brazil (14.7%) [5,15]. It was close to the incidence rate in New York State, which was 8.2% in 2009 and the nationwide U.S. average, as reported by 246 medical centers throughout the country 2005 (3.8-10.6%) [3,16]. China is a developing country. It covers a vast territory and a wide range of economic and cultural development. Medical conditions can vary considerably in different regions, especially between southeastern coastal cities and the remote northwest. This may be the reason why the incidence of LBW and perinatal outcomes in these regions is also different. The incidence of LBW in Southwestern China was found to be the highest in the country. These data were mostly collected from the Guangdong Province. Due to geographical, genetic, and dietary factors, the average height in the Guangdong province is lower than the national average in both men and women, and most Cantonese individuals tend to be thin. This may be related to the high incidence of LBW in this region. Upon further analysis, the incidence of LBW in Beijing and Shanghai was found to be similar to that in developed countries. However, the incidence of LBW in Xinjiang, which is a remote and highly rural region, was found to be significantly higher than in economically developed cities. This trend suggests that the occurrence of LBW is related to economic development, which is consistent with the results of studies carried out in other parts of the world [17]. In economically underdeveloped regions, the level of health care is relatively low, and pregnant women commonly do not receive adequate prenatal care and poor nutrition. This increases the risk of pregnancy complications. Low levels of medical treatment also leave patients and doctors with insufficient means of diagnosing, preventing, and treating LBW.

In China, hospitals are managed on a class system based on the level of medical care provided and on personnel and equipment. In the present study, analysis of the incidence of LBW in hospitals of different classes revealed rather large differences. In tertiary care hospitals, that have medical equipment that is relatively up-to-date with health care that is of relatively good quality, the incidence of LBW was found to be relatively high, accounting for 90% of all cases of LBW. In secondary care hospitals, the incidence of LBW incidence was lower than in tertiary care hospitals. This suggests that differences in medical equipment and levels of hospitals in different classes may be relevant. The majority of PTB infants with LBW are transferred to teriary care hospitals where the quality of medical treatment is relatively high, and some women with high-risk pregnancies or pregnancy complications are also transferred to teriary care hospitals.

LBW is caused by two leading factors in China, PTB and intrauterine growth restriction. In developed countries, it is caused mainly by PTB; however, in developing countries the main cause is intrauterine growth restriction [14]. In 2000, 38.8% of all cases of LBW in China were due to PTB, and 61.2% were due to intrauterine growth restriction [8]. The results of the present study showed that more than one third LBW cases were due to PTB. This suggests that the causes of LBW in China have changed over the past 10 years. This is not surprising. By upgrading facilities, strengthening the training of personnel and establishing a referral network for critically ill premature children, the neonatal intensive care units (NICUs) have been able to substantially increase the survival rate of infants with extremely low birth weights. The successful manual intervention is started 34–30 weeks in advance, allowing the treatment of preterm infants at an early gestational age. China’s NICUs have undergone continuous improvement, resulting in a substantial increase in the survival rates of LBW and PTB infants. Medical interventions for high-risk pregnant women have also improved. The continuous development of new assisted reproductive technology has increased the number of twins and multiple pregnancies, which also leads to an increased incidence of PTB. More attention has been paid to the health care of women during pregnancy, and high-risk pregnancy management is emphasized. Early diagnosis and interventional treatment have become available to treat pregnancy comorbidities and complications. Attention has also been paid to intrauterine growth restriction, and the corresponding treatment during pregnancy and prognosis have been improved. These changes have in turn changed the proportions of causes of LBW, making them more similar to those found in developed countries.

LBW is caused by both direct factors such as pregnancy complications and by indirect factors such as socio-economic development. The factors that contribute to LBW differ across different countries and across different regions within certain countries. Previous studies have reported that maternal age, level of education, race, occupation, family financial status, height, weight, smoking, drinking, health care during pregnancy, history of disease, pregnancy comorbidity, complications, and other factors are all related to LBW [5,14]. The present study showed that factors that correlated with LBW occurrence included level of education, age less than 20 years or greater than 35 years, and past history of miscarriage or pregnancy comorbidities and complications, such as GDM, hypertensive disorders during pregnancy, anemia, oligohydramnios, and similar conditions. The maternal level of education was found to be significantly closely correlated with the occurrence of LBW. The lower the level of maternal education, the higher the incidence of LBW. Because of the continuing socio-cultural development and the enforcement of compulsory education in China, pregnant women with low levels of education are mostly located in remote, economically underdeveloped regions. They tend to be uninformed regarding pregnancy nutrition and health care, and local levels of health care also tend to be poor, preventing them from undergoing regular checkups during pregnancy, which results in an increase in the incidence of LBW. The results of the present study showed the maternal age, both ≤20 years and ≥35 years, to be a risk factor for LBW. When pregnant women are too young, risk factors include incomplete development of organs and tissues, unmarried status, low level of education, poor financial conditions, and low body weight, which can lead to notable increases in the incidence of LBW. Some studies have shown that among pregnant teenagers 10–19 years of age, the incidence of LBW was significantly higher than among adult pregnant women [15,18]. Among 20,560 LBW mothers in New York City, pregnant teenagers accounted for 8.4%, and each LBW cost an average of 51,600 U.S. dollars. Considerable attention should be paid to pregnancy among teenagers [19-21]. However, as women age, different bodily functions gradually become less efficient, and the risks of chronic conditions and of pregnancy complications increase. Many studies have shown that the incidence of perinatal complications among pregnant women of advanced age is significantly higher than among younger women [22]. These complications can increase the incidence of LBW. A previous history of miscarriage, PTB, fetal death, or stillbirths were also found to be risk factors for LBW, which is consistent with previous reports [23]. The results of the present study also showed the incidence of LBW among pregnant women with obstetric comorbidities and complications to be higher than among those without such complications. PTB is an important cause of LBW. Hypertensive disorders in pregnancy are also important contributors to LBW. The incidence of LBW among pregnant women with hypertensive diseases was found to be 5 times that of other pregnant women, Hypertention leads to insufficient blood flow to the placenta and limits fetal development. If the disease progresses further, or if fetal distress occurs, the pregnancy must end early, and these iatrogenic preterm births increase the incidence of LBW. This problem should be addressed and should be considered high-risk pregnancies, simialar to pregnancies in women with hypertensive disorders. Premature rupture of membranes may cause intrauterine infection, eventually leading to LBW. The results showed the incidence of LBW among pregnant women with oligohydramnios to be twice that of other women. A study by Coutinho also showed a correlation between oligohydramnios, LBW, and adverse outcomes [24]. Anemia during pregnancy was also found to be a risk factor for LBW. In 2009, the WHO stated that iron deficiency is a worldwide nutritional problem. A recent study carried out in Mexico showed that 20.6% of pregnant women suffered from anemia and their risk of developing LBW and PTB were significantly higher than in of non-anemic women [25]. Therefore, active prevention, early diagnosis, and timely treatment of obstetric complications are especially important. The present study showed that a low BMI before pregnancy (<18.5) increased the incidence of LBW. Women with low BMIs may be malnourished, increasing their chance of giving birth to LBW infants. The present study also showed GDM and high BMI before pregnancy to be protective factors, associated with lower incidences of LBW. Current studies have shown that the worldwide occurrence of GDM in recent years has increased significantly. Obesity, higher BMI and too much weight gain during pregnancy are all risk factors for GDM, and uncontrolled GDM can significantly increase rates of macrosomia, birth injury, and cesarean sections [26,27]. Therefore, there should be a balance between pre-pregnancy weight, weight gain during pregnancy, and birth weight, so that excess weight and weight gain are avoided but the chance of LBW remains low. The majority of studies carried out in counties other than China have shown that smoking and drinking lead to increases in the incidence of LBW [15,21]. However, the present study showed no correlation between smoking or drinking and LBW. This may be related to the fact that far fewer Chinese women than western women smoke and drink alcohol.

Pregnancy outcomes of full-term LBW, and PTB and LBW are two major causes of adverse perinatal outcomes, and PTB is the major cause of perinatal deaths. Each year approximately 3 million neonates die of PTB worldwide [28]. When preterm infants with a gestational age of 34 weeks weighing 2400 g were compared to full-term infants of the same weight, the latter were classified as small for gestational age (SGA), meaning their weights were below the 10th percentile for their gestational age. SGA is a good indicator of neonatal morbidity and mortality. The average birth weight in the Chinese population at a gestational age of 37 weeks is 2922 g, and the cutoff for the 10th percentile is 2413 g. Therefore, it is important to pay great attention to full-term LBW infants. In the present study, the influence of PTB was removed and pregnancy outcomes of full-term LBW alone were analyzed. In recent years, the proportion of childbirths involving cesarean sections has increased considerably worldwide, and it is significantly higher in China than in western countries. LBW is an important indicator for the need of a cesarean section. The results of the present study show the rate of cesarean section among LBW infants to be significantly higher than among neonates with normal birth weights. Of the cesarean sections, emergent cesarean sections are significantly more common among full-term LWB neonates than full-term normal-weight neonates. Among full-term LBW neonates, the rate of cesarean sections due to fetal distress in utero and placental abruption secondary to limited fetal growth, other complications, or inability of the fetus to tolerate a vaginal delivery is relatively high. As reported by Coutinho, the rate of cesarean sections among LBW infants was 2.4 times that infants of normal birth weight, and the rate of converting to cesarean section during delivery among LBW infants was 1.5 times that of infants of normal birth weight [24]. The present study analyzed the correlation between different delivery methods among full-term LBW infants and their prognoses. The incidences of stillbirths, neonatal complications and neonatal deaths among LBW infants delivered by cesarean section were lower than those delivered vaginally. This is consistent with results reported by Coutinho, who found cesarean sections to be safer for LBW neonates than vaginal birth [24]. The present study compared the outcomes of full-term LBW infants and full-term infants with normal birth weights. The incidence of severe neonatal asphyxia, neonatal complications, and neonatal death among full-term LBW infants was significantly higher than among full-term infants with normal birth weights. This suggests that full-term LBW can lead to adverse pregnancy outcomes, which is consistent with previous reports [28].

Regarding the prognosis of LBW, with the development of perinatology, the survival rate of LBW infants has substantially increased. However, some adverse outcomes are still relatively common, especially long-term complications such as cerebral palsy, delayed neurodevelopment, and visial and hearing impairments. These all have adverse effects on adults and their offspring. For these reasons, LBW merits considerable attention. To reduce the incidence of LBW, increases in nutrient intake before conception, proper pregnancy education, and good health care during pregnancy should be fostered, and high-risk factors for LBW should be avoided. This may allow LBW to be prevented or at least diagnosed early and treated properly. In addition, comprehensive assessment of the mother and child situation should be made to allow the selection of a proper delivery method, thus improving the prognosis of LBW neonates.

## Conclusions

The incidence of LBW in mainland China is currently higher than that reported in 2000. It is lower in economically developed cities and higher in remote, underdeveloped regions. The incidence of LBW is closely correlated with the maternal level of education, age, past history of adverse pregnancies, and pregnancy comorbidities and complications. The prognosis of full-term LBW infants is significantly poorer than that of full-term infants with normal birth weights. Therefore, the incidence of LBW is an important measure of social development, and maternal and child health care. Prevention and treatment of LBW infants are key issues in the fields of eugenics and perinatology. To improve survival and quality of life in developing countries it is crucial to reduce the incidence of LBW.

## Competing interests

The authors declare that they have no competing interests.

## Authors’ contributions

YC performed the statistical analysis and prepared the manuscript. WYZ participated in the design and coordination of the study and revised the manuscript. YR and LYZ carried out the data collection. GHL and XW were partially involved in analyzing the data. All authors read and approved the final manuscript.

## Pre-publication history

The pre-publication history for this paper can be accessed here:

http://www.biomedcentral.com/1471-2393/13/242/prepub

## Supplementary Material

Additional file 1 **The additional file is questionnaire of this paper.** The questionnaire was designed by obstetric and statistical experts, it included pregnant basic information, history of abnormal pregnancy, pregnancy complications and comorbidities, intrapartum and postpartum, and neonates information. Click here for file
