# Supplementary material for: An epidemiological survey on low birth weight infants in China and analysis of outcomes of full-term low birth weight infants
Source: BMC Pregnancy Childbirth. 2013 Dec 26;13:242. doi: 10.1186/1471-2393-13-242 (PMC3877972; doi:10.1186/1471-2393-13-242)
Supplement: Additional file 1 — The additional file is questionnaire of this paper. The questionnaire was designed by obstetric and statistical experts, it included pregnant basic information, history of abnormal pregnancy, pregnancy complications and comorbidities, intrapartum and postpartum, and neonates information. [file 1471-2393-13-242-S1.docx]

# Additional files

**Additional file 1 –** **questionnaire**

Investigation Date：

Delivery gestational age weeks delivery date:

Section One Pregnant Basic Information

[admission](link:admission) [number](link:number) Age Her husband's age

1. Mound outside residents type □ 1. urban 2. rural

2. education level □ 1. Tertiary or higher Technical

2. secondary school, high school, or higher

3. Middle school and below

4. Illiterate

3. Smoking □ 0. No 1. Yes

4. Drinking □ 0.No 1. Occasionally 2. Often at times

5. Pregnant time □ parity □ abotion □ survival

History of abnormal pregnancy：

□Spontaneous abortion

□premature

□[intrauterine](link:intrauterine) [death](link:death)

□stillbirth

□[ectopic](link:ectopic) [gestation](link:gestation)

□Deformed child birth history

□early neonatal death

□multiple pregnancies

6. The way of pregnancy □ 1. natural conception

2. IVF-ET

3.Other artificial assisted reproduction

7. Family history □ 0.No

1.Yes（Hypertensive，GDM，Congenital

Disease，ICP，others）

2. [not](link:not) [quite](link:quite) [clear](link:clear)

8. previous history □ 0.No

1.Yes （Hypertensive，kidney disease，

Chronic hypertension，GDM，Heart

disease，the immune system disease，

ICP，others）

2. [not](link:not) [quite](link:quite) [clear](link:clear)

9. height cm

Prepregnancy /early pregnancy weight kg

Prepregnancy /early pregnancy blood pressure / mmHg

Ante Partum weight kg

10. Pregnant women blood type：ABO □ 1.A 2.B 3.AB 4.O

Rh □ 1. Positive 2. negative

11. HBV：HBsAg □ HBsAb □ HBcAg □ HBcAb □ HBeAb

[hepatitis](link:hepatitis)[B](link:B)DNA (<103copies/ml;

103-105copies/ml;

>105copies/ml;

Not Conducted）

12. Hospital stay days

Hospitalization Expenses ¥

Pregnancy complications and comorbidities

13. PROM □ 0.No

1.Yes (if 0, skip the following content)

gestational weeks weeks AFI cm or AF cm

Rupture early diagnosis way (can choose more)

□ 1.clinical

2. Insulin-like factor test

3. Amniotic fluid crystallization

4. Vaginal pH

bed rest □ 0.No 1Yes

[antibiotic](link:antibiotic) [use](link:use) □ 0.No 1.Yes（Penicillins, cephalosporins, other）

[medication](link:medication) [time](link:time) □ 1.<6h 2.6-12h 3. 12-24h 4.>24h

clinical monitoring

T ℃ HR 次/分 WBC ×109/L GR %

CRP □ 0.No 1. mg/L

FHR □ 1.>160bpm 2.<160bpm CST □1. Positive 2. negative

14. premature birth □ 0.No

1. Yes (if 0, skip the following content)

Whether miscarriage treatment□ 0. No 1. Yes

Medicine □ 1. ritodrine Hydrochloride

2. [magnesium](link:magnesium) [sulfate](link:sulfate)

3. Atosiban Acetate

4. others

Therapy to promote fetal lung development □ 0. No 1.Yes

[etiological](link:etiological) [examination](link:examination) BV □ 0.not done1.

1. Positive 2. negative

mycete □0.not done，1.do 1. Positive 2. Negative

mycoplasma □0.not done，1.do 1. Mh Positive 2. UU Negative

chlamydia □0.not done，1.do 1. Positive 2. Negative

germiculture □0.not done，1.do 1. Positive 2. Negative

Ultra cervical length cm Detecting gestational age weeks

fFN □0.not done, 1.do 1. Positive 2. Negative

Neonatal outcome 1 □1.rooming

2. Transferred to NICU

3.death

4. intrauterine fetal death

5. others

Neonatal outcome 2 □ 1.rooming

2. Transferred to NICU

3.death

4. intrauterine fetal death

5. others

15. GDM □ 0. No

1. Yes

2.pregestational diabetes mellitus

(if 0, skip the following content)

INS Initial detection of gestational age Detection of gestational last

CP Initial detection of gestational age Detection of gestational last

Treatment ( check all that apply )

No treatment

Treatment 1. Nutrition

2. drugs (oral medications, insulin)

HbA1c Initial detection of gestational age ％

Detection of gestational last ％

16. [hypertensive](link:hypertensive) [disorder](link:disorder) [complicating](link:complicating) [pregnancy](link:pregnancy)

□ 0. No

1. Yes (if 0, skip the following content)

classify □ 1. gestational hypertension

2. mild preeclampsia

3. severe preeclampsia

4. eclampsia

5. Chronic hypertension concurrency preeclampsia

6. Chronic hypertension combined pregnancy

Diagnosis of gestational age（w）

edema □ 0.No 1.+ 2.++ 3.+++ 4.++++

Protein □ 1.negative 2.+ 3.++ 4.+++

[urine](link:urine) [protein](link:protein) [quantitation](link:quantitation) g/24h

total plasma protein g/L [plasma albumin](link:plasma-albumin) g/L

[hypoproteinemia](link:hypoproteinemia) □ 0.No 1.Yes

Whether the complications □ 0.No 1.Yes（HELLP syndromic，[acute](link:acute) [renal](link:renal) [failure](link:failure)，DIC，FGR，Placental abruption, heart failure, pulmonary edema, cerebrovascular accident，others）

17.fetal growth restrict（FGR）

□ 0.No

1. Yes (if 0, skip the following content)

Diagnosis of gestational age weeks HCT %

ultrasound results：BPD cm FL cm AFI cm或AF cm HC cm AC cm S/D

Laboratory Examinations

D-D ng/ml Detecting gestational age weeks

KB % Detecting gestational age weeks

CVT： Peripheral resistance blood viscosity

Detecting gestational age weeks

TORCH □0.not done, 1.do 1. Positive 2. Negative

Positive project（Rubella, toxoplasma, coxsackie, giant cells,

single blister）Detecting gestational age weeks

B19 □0.not done, 1.do 1. Positive 2. Negative

Detecting gestational age weeks

treatment □ 0. No treatment

1. nutrition

2. [dilatation](link:dilatation)

3. nutrition & [dilatation](link:dilatation)

4. others

18. [fetal](link:fetal) [distress](link:distress) □ 0.No

1. Amniotic fluid type

2.fetal heart type

3. [mixed](link:mixed) [type](link:type)

19. anaemia □ 0.No 1.Yes （if 0, skip the following content）

type □ 1. Iron deficiency anemia

2. gigantic young cell anemia

3. Mediterranean anemia

4.others

Detecting gestational age weeks

HGB g/L HCT % PLT ×109/L MCH pg MCV fL MCHC g/L

20. Intrahepatic cholestasis of pregnancy

□0.No

1.Yes （if 0, skip the following content）

Total bile acid umol/L ; Cholic acid umol/L

ALT IU/L AST IU/L

Wether the itch □ 0. No 1.Yes Detecting gestational age

liver ultrasound □ 0.not done

1.negative

2. [cholelithiasis](link:cholelithiasis)

3.others

treatment □ 1. temporization 2.medicine 3.not treatment

Way to terminate a pregnancy □ 1. [induced](link:induced) [labour](link:labour)

2.C-section

3. Natural labor

21. Heart disease during pregnancy

□ 0.No

1. Preconception exists（Congenital heart disease; Rheumatic heart disease；others）

2. Prenatal diagnosis（Congenital heart disease; Rheumatic heart disease；peripartum cardiomyopathy ；others）

Detecting gestational age weeks

[cardiac](link:cardiac) [functional](link:functional) [grading](link:grading)

□ 0. No classification

1.Ⅰ

2.Ⅱ

3.Ⅲ

4.Ⅳ

22. acute fatty liver of pregnancy

□ 0. No

1. Yes

2. dignosis in [pregnancy](link:pregnancy) [period](link:period)

Detecting gestational age weeks

23. Kidney disease in pregnancy

□ 0. No

1. Preconception exists

2.dignosis in [pregnancy](link:pregnancy) [period](link:period)

Detecting gestational age weeks

24. thyroid disorders in pregnancy

□ 0. No

1. Preconception exists

（Hyperthyroidism; Thyroid function decrease; Hashimoto's

disease; others）

2. dignosis in [pregnancy](link:pregnancy) [period](link:period)

（Hyperthyroidism; Thyroid function decrease; Hashimoto's

disease; others） Detecting gestational age weeks

whether treatment

□ 0.No

1. Thyroxine tablet

3.others

25. Diseases of the blood system during pregnancy

□0.No

1. Preconception exists（Aplastic anemia; The Mediterranean

anemia; Idiopathic thrombocytopenia. others）

2. dignosis in

[pregnancy](link:pregnancy) [period](link:period)（Aplastic anemia; The Mediterranean anemia;

Idiopathic thrombocytopenia. others）

Detecting gestational age weeks

26. Diseases of the immune system during pregnancy

□0.No

1. Preconception exists（SLE；For antiphospholipid antibody syndrome;

Sjogren's syndrome. others）

2. dignosis in [pregnancy](link:pregnancy) [period](link:period)（SLE；For antiphospholipid antibody syndrome;

Sjogren's syndrome. others）

Detecting gestational age weeks

27. Genital tract malformation

□ 0.No

1. [uterus](link:uterus) [unicornis](link:unicornis)

2 [uterus](link:uterus) [bicornis](link:bicornis)

3. [uterus](link:uterus) [duplex](link:duplex)

4 uterus mediastinum

5. longitudinal vaginal septum

28. pregnancy associated with hysteromyoma

□ 0.No

1.Yes （if 0, skip the following content）

fibroids size □ a.≤2cm b.2-5cm c.>5cm

Number of fibroids □ a.single b. [pilosity](link:pilosity)

location □1. [corpus](link:corpus) [uteri](link:uteri)

2.cervix

3. [lower](link:lower) [uterine](link:uterine) [segment](link:segment)

□ a. subserous b. submucosal c. intrmurl myom

Whether intraoperative □ a.Yes b.No

29. Abnormal amniotic fluid

□ 0.No

1. [polyhydramnios](link:polyhydramnios)（AFI≥18cm or AF≥8cm）

2. [oligoamnios](link:oligoamnios)（AFI≤5cm或AF≤2cm，）

more than two ultrasonic diagnosis

[FBG](link:FBG) ( [fasting](link:fasting) [blood-glucose](link:blood-glucose) )

30. TTTS

□ 0.No

1.Yes（monochorionic, di-amniotic，[not](link:not) [quite](link:quite) [clear](link:clear)）

（if 0, skip the following content）

Detecting gestational age weeks [amniotic](link:amniotic) [fluid](link:fluid) [volume](link:volume)（AF）：

The first baby cm The second baby cm

TTTs Ultrasonic classification

□1. levelⅠ

2. levelⅡ

3. Level Ⅲ

4. Level Ⅳ

5. [not](link:not) [quite](link:quite) [clear](link:clear)

treatment

□ 0.No

1. amnioreduction amniodrainage

2. Laser coagulation

3. multifetal pregnancy reduction

4. Amniotic cavity chorionic colostomy

5. [radio](link:radio) [frequency](link:frequency)

6.others

Intrapartum and postpartum

31. mode of delivery

□ 1. caesarian section

2. Forceps

3. vacuum extraction.

4. Normal Vaginal Delivery

5. assisted breech delivery

6. [breech](link:breech) [extraction](link:extraction)

Fetal position and Fetal lie

1. [position](link:position) [of](link:of) [the](link:the) [head](link:head)（occipitoanterior position，persistent occipital transverse position，persistent occipitoposterior position ，face presentation，mentum prese，ntation，head sincipitoposterior position，anterior asynclitism）

2. [breech](link:breech) [presentation](link:presentation)

3. [transverse](link:transverse) [presentation](link:presentation)

32. Cesarean section indication □（Multiple choices）

□ 1. no indication

2. Maternal factors

（Pelvic abnormalities, abnormal labor, placental abruption, placenta previa, heart disease with the pregnancy, heart failure, pregnancy with acute fatty liver, oligohydramnios, severe preeclampsia, scar uterus, combination of uterine fibroids, ovarian cyst first-timer head floating, high myopia, older first-timer, genital tract infection）

3.Fetal factors（Precious fetus, fetal distress and fetal blood

transfusion syndrome, macrosomia, FGR, abnormal position, multiple pregnancies, umbilical cord prolapse）

4. others

33. Cesarean delivery time

□ 1. Elective

2.emergency

Complications after cesarean section

□ 0.No

1. [infection](link:infection) [of](link:of) [incisional](link:incisional) [wound](link:wound)

2. Incision delayed crack

3. postpartum morbidity

4. [deep](link:deep) [venous](link:venous) [thrombosis](link:thrombosis)

34. Vaginal midwifery indications

Maternal factors （uterine atony，To prevent the second prolonged labor，prolonged labor，Pregnant women heart disease，pregnancy-induced hypertension，GDM，persistent occipital transverse position，persistent occipitoposterior position）

Fetal factors（Fetal distress, umbilical cord prolapse）

others

35. Forceps way：

□ 1. Export Position Forceps

2. Low forceps

3. In high forceps

4. Coriolis forceps

36. lateral episiotomy □ 0. No 1. Yes

37. labor analgesia □ 0. No 1. Yes

Labor analgesia method

□ 1.combined spinal-epidural anesthesia

2. Doula labor

3. water birth

4. Intravenous Anesthesia

5. Physical pain

38. placenta 1 shape

□ 0.normal

1. battledore placenta

2. velamentous placenta

3. placenta circumvallata

4. Placenta succenturiata 5.others

placenta weight g placenta size × × cm

placenta location □1. Anterior uterine wall

2. posterior uterine wall

3.fundus

4. [lower](link:lower) [uterine](link:uterine) [segment](link:segment)

5. [not](link:not) [quite](link:quite) [clear](link:clear)

39. placenta previa □ 0.No 1. Yes（marginal，partial，complete）

40. placental abruption □ 0.No 1. Yes

41. [placenta](link:placenta) [implantation](link:implantation) □ 0.No 1.Yes

[adherent](link:adherent) [placenta](link:placenta) □ 0.No 1.Yes

[adherent](link:adherent) [placenta](link:placenta) □ 0.No 1.Yes

42. placental pathology

□ 0.not done

1. Acute inflammation of chorionic

2. Chronic chorionic inflammation of chorionic

3. Acute umbilical cord inflammation

4.others

43. characteristics of the amniotic fluid

□ 0.clear

1. contamination of amniotic fluid 1. Ⅰdegree

2.Ⅱdegree

3.Ⅲ degree

4.bloody

44. postpartum hemorrhage

□ 0.No 1.Yes

（if 0, skip the following content）

The amount of postpartum bleeding ml

[measuring](link:measuring) [method](link:method)

□ 1. Visual method

2. Weighing method

3. Volume method

4. According to the estimation of hemoglobin

causes of hemorrhage

1. uterine atony

2. soft birth canal injury

3.Placenta and membranes residue

4.Blood Coagulopathy dyfunction

Secondary coagulant function abnormality

□ 0.No 1.Yes

special treatment

□ 0.No

1. B-lynch sew up

2. Uterine cavity filling yarn

3. Intrauterine water capsule filling pressure

4. Uterine artery ligation

5. interventional therapy

6. Hysterectomy

7. Drug therapy

8.others

[transfusion](link:transfusion) □ 0.No 1.Yes

the amount of blood transfusion：Red Blood Cells Suspension

Plasma whole blood platelet cold precipitation

45. [amniotic](link:amniotic) [fluid](link:fluid) [embolism](link:embolism) □ 0.No 1. Yes

46. pulmonary embolism □ 0.No 1. Yes

47. Maternal outcomes □ 1.fine

2. complications

3.death: cause of death

[laboratory](link:laboratory) [examination](link:examination)

48. [blood](link:blood) [routine](link:routine) [examination](link:examination)

First trimester HB g/L HCT % PLT ×109/L

second trimester HB g/L HCT % PLT ×109/L

third trimester HB g/L HCT % PLT ×109/L

post partum HB g/L HCT % PLT ×109/L

49. Blood sugar test report

[FBG](link:FBG) ( [fasting](link:fasting) [blood-glucose](link:blood-glucose) ) mmol/L

Detecting gestational age weeks

50gGCT mg/dl Detecting gestational age weeks

OGTT report（mmol/L）：FBG 1h 2h 3h

Detecting gestational age weeks

50. The last time of prenatal ultrasound diagnosis gestational weeks

BPD cm FL cm HC cm AC cm AFI cm S/D

single umbilical artery □ 0.No 1.Yes

Section Neonates

51. Neonatal 1 basic situation

Sex Weight（g） Height（cm） Apgar Score（1-5-10min）

Malformation fetus or neonate

□ 0. No

1. chromosome（trisomy 21 sydrome，trisomy 18 sydrome， trisomy 13 sydrome，others）

2. NTD（neural tube defects）

3. Congenital heart disease

4. Cleft

5. small ears or no ears

6. Esophageal stenosis or atresia

7. anal atresia

8. hypospadias

9 . Bladder exstrophy

10. equinus varus

11. multi-fingered

12. Syndactyly

13. Limbs short shrink

14. congenital diaphragmatic hernia

15. omphalocele

16. gastroschisis

17.others

Neonatal Complications

□ 0.No

1. hypoglycemia

2 . Hypocalcemia

3 neonatal respiratory distress syndrome

4. wet lung

5. Pneumonia

6. Jaundice

7. polycythemia

8. birth injury ( skeletal neuromuscular )

9. Infection

10.others

The length of the umbilical cord（cm） [cord](link:cord) [entanglement](link:entanglement)

Neonates outcomes □ 1.normal

2.complication

3. Deformities

4. Death cause of death

52. Neonatal 2 basic situation

Sex Weight（g） Height（cm） Apgar Score（1-5-10min）

Malformation fetus or neonate

□ 0. No

1. chromosome（trisomy 21 sydrome，trisomy 18 sydrome， trisomy 13 sydrome，others）

2. NTD（neural tube defects）

3. Congenital heart disease

4. Cleft

5. small ears or no ears

6. Esophageal stenosis or atresia

7. anal atresia

8. hypospadias

9 . Bladder exstrophy

10. equinus varus

11. multi-fingered

12. Syndactyly

13. Limbs short shrink

14. congenital diaphragmatic hernia

15. omphalocele

16. gastroschisis

17.others

Neonatal Complications

□ 0.No

1. hypoglycemia

2 . Hypocalcemia

3 neonatal respiratory distress syndrome

4. wet lung

5. Pneumonia

6. Jaundice

7. polycythemia

8. birth injury ( skeletal neuromuscular )

9. Infection

10.others

The length of the umbilical cord（cm） [cord](link:cord) [entanglement](link:entanglement)

Neonates outcomes □ 1.normal

2.complication

3. Deformities

4. Death cause of death

Section Three each growth and development indexes

The gestational weight（kg）

12w 13w 14w 15w 16w 17w

18w 19w 20w 21w 22w 23w

24w 25w 26w 27w 28w 29w

30w 31w 32w 33w 34w 35w

36w 37w 38w 39w 40w 41w 42w

Initial inspection gestational age weeks

The number of prenatal care

Prenatal fetal ultrasound examination results（cm）

[biparietal](link:biparietal) [diameter](link:diameter)（BPD）

12w 13w 14w 15w 16w 17w

18w 19w 20w 21w 22w 23w

24w 25w 26w 27w 28w 29w

30w 31w 32w 33w 34w 35w

36w 37w 38w 39w 40w 41w 42w

femur length（FL）

12w 13w 14w 15w 16w 17w

18w 19w 20w 21w 22w 23w

24w 25w 26w 27w 28w 29w

30w 31w 32w 33w 34w 35w

36w 37w 38w 39w 40w 41w 42w

[head](link:head) [circumference](link:circumference)（HC）

12w 13w 14w 15w 16w 17w

18w 19w 20w 21w 22w 23w

24w 25w 26w 27w 28w 29w

30w 31w 32w 33w 34w 35w

36w 37w 38w 39w 40w 41w 42w

[abdominal](link:abdominal) [girth](link:girth)（AC）：

12w 13w 14w 15w 16w 17w

18w 19w 20w 21w 22w 23w

24w 25w 26w 27w 28w 29w

30w 31w 32w 33w 34w 35w

36w 37w 38w 39w 40w 41w 42w

[umbilical](link:umbilical) [artery](link:artery) [flow](link:flow)（S/D）：

12w 13w 14w 15w 16w 17w

18w 19w 20w 21w 22w 23w

24w 25w 26w 27w 28w 29w

30w 31w 32w 33w 34w 35w

36w 37w 38w 39w 40w 41w 42w
